# Neuromodulatory Effects of Substantia Nigra Pars Reticulata Deep Brain Stimulation (SNr-DBS) in the 6-Hydroxydopamine Rat Model of Parkinson’s Disease

**DOI:** 10.3390/medicina62040714

**Published:** 2026-04-09

**Authors:** Eylem Turgut, Hande Parlak, Pinar Eser, Yasin Temel, Ali Jahanshahi, Levent Sarıkcıoglu, Gamze Erguler Tanrıover, Tanju Ucar, Ersoy Kocabicak, Aysel Agar

**Affiliations:** 1 Department of Physiology, Faculty of Medicine, Akdeniz University, 07070 Antalya, Turkey; eylemtelli@yahoo.com (E.T.); handeparlak@akdeniz.edu.tr (H.P.); ayagar@akdeniz.edu.tr (A.A.); 2 Department of Physiology, Faculty of Medicine, Amasya University, 05000 Amasya, Turkey; 3 Department of Neurosurgery, Faculty of Medicine, Bursa Uludag University, 16059 Bursa, Turkey; pinarocak@uludag.edu.tr; 4 Department of Neurosurgery, Maastricht University Medical Center, 6202 AZ Maastricht, The Netherlands; y.temel@maastrichtuniversity.nl (Y.T.); a.jahanshahi@maastrichtuniversity.nl (A.J.); 5 Netherlands Institute for Neuroscience, Royal Netherlands Academy of Arts and Sciences, 1105 BA Amsterdam, The Netherlands; 6 Department of Anatomy, Faculty of Medicine, Akdeniz University, 07070 Antalya, Turkey; levent@akdeniz.edu.tr; 7 Department of Histology and Embryology, Faculty of Medicine, Akdeniz University, 07070 Antalya, Turkey; gamzetanriover@akdeniz.edu.tr; 8 Department of Neurosurgery, Istanbul Atlas University, 34408 Istanbul, Turkey; tnjucr@gmail.com

**Keywords:** Parkinson’s disease, deep brain stimulation, globus pallidus internus, substantia nigra pars reticulata, subthalamic nucleus, 6-hydroxydopamine

## Abstract

*Background and Objectives:* Parkinson’s disease (PD) is a neurodegenerative disorder marked by bradykinesia, rigidity, and tremor. While deep brain stimulation (DBS) of the subthalamic nucleus (STN) and globus pallidus internus (GPi) effectively alleviates motor symptoms, the potential of targeting the substantia nigra pars reticulata (SNr) is less understood. This study investigates the effects of mid-term DBS of the SNr on motor function and neuroplasticity in a 6-hydroxydopamine (6-OHDA) rat model of PD. *Methods:* Adult male Sprague-Dawley rats (280–300 g) were divided into healthy control (n = 10), PD (n = 9), sham-DBS (n = 7), and SNr-DBS (n = 7) groups. Bilateral striatal 6-OHDA lesions induced PD. High-frequency (130 Hz, 60 µs) SNr-DBS was delivered for 14 days. Locomotor activity (open-field), gait (footprint method), and motor coordination (rotarod) were assessed. Tyrosine hydroxylase (TH) expression in the SN and c-Fos and BDNF expression in the cerebellum, prefrontal cortex (PFC), and ventrolateral thalamus were analyzed histologically. *Results:* SNr-DBS significantly improved ambulation and horizontal activity compared to the PD group (*p* < 0.05). Gait analysis showed significant improvements in forelimb/hindlimb stride length and stance width, while rotarod performance indicated enhanced motor coordination (*p* < 0.05). Histology revealed increased TH expression in the SN and elevated c-Fos and BDNF levels in the cerebellum, PFC, and thalamus in the SNr-DBS group vs. PD rats (*p* < 0.05). *Conclusions:* Mid-term SNr-DBS produced significant functional gains in motor activity and coordination in a 6-OHDA PD model, together with molecular evidence of dopaminergic enhancement and neuroplastic activation. These translational findings suggest that targeting the SNr may offer a clinically relevant alternative for patients with PD, particularly for those who may not optimally respond to conventional STN or GPi stimulation.

## 1. Introduction

Parkinson’s disease (PD) is a progressive neurodegenerative disorder characterized by the loss of dopaminergic neurons in the nigrostriatal pathway. Clinically, PD presents with motor symptoms like tremor, rigidity, bradykinesia, and postural instability, along with non-motor symptoms including cognitive impairment, emotional disturbances, and autonomic dysfunction [1,2]. While no disease-modifying pharmacological treatments are currently available, dopamine-based therapies remain the cornerstone for managing motor symptoms [3]. In cases complicated by medication-resistant tremor, dyskinesia, or the “wearing-off” phenomenon, advanced device-assisted therapies may be necessary [4].

Deep brain stimulation (DBS) of the subthalamic nucleus (STN) and internal globus pallidus (GPi) is an established therapy for advanced PD with motor complications, supported by large randomized trials [5,6,7]. However, the optimal target remains debated, with STN-DBS facilitating greater medication reduction [8] and GPi-DBS linked to improved functioning and reduced dyskinesia [9,10]. Despite these benefits, axial symptoms like gait and balance impairment worsen over time in up to 80% of advanced PD patients, complicating treatment outcomes [11,12]. In STN-DBS patients, using a more ventral contact to target the SN pars reticulata (SNr) has been suggested [13,14] to improve gait-related issues, in addition to preventing current spread to the internal capsule [13] and lowering frequency [15].

The SNr, an area implicated in motor control, has recently emerged as a potential DBS target for PD, particularly in addressing gait and balance dysfunction. Though clinical evidence supports its efficacy in improving locomotor symptoms, experimental studies on SNr stimulation in PD are still limited. This study aims to investigate the effects of high-frequency SNr-DBS (14 days) on locomotion in the 6-hydroxydopamine (6-OHDA) parkinsonian rat model. We focus on evaluating tyrosine hydroxylase (TH) expression in the substantia nigra pars compacta (SNc), c-Fos, and brain-derived neurotrophic factor (BDNF) expression as markers of potential neuroprotection and neuronal activation in the cerebellum, prefrontal cortex (PFC), and ventrolateral thalamus, to better understand the neurophysiological effects of SNr-DBS.

## 2. Materials and Methods

### 2.1. Subjects and Experimental PD Model

Adult male Sprague-Dawley rats (280–300 g; n = 33) were housed in controlled cages with a constant temperature and a 12 h light–dark cycle, with food and water provided ad libitum. All procedures were approved by the Akdeniz University Experimental Animals Local Ethics Committee (01.03.2016; Protocol No: 2016.03.08) and were conducted in accordance with the National Institutes of Health Guide for the Care and Use of Laboratory Animals. All experimental details were reported in compliance with the ARRIVE guidelines.

To induce the PD model, animals were anesthetized with xylazine (10 mg/kg) and ketamine (90 mg/kg), then placed in a stereotaxic apparatus. Bilateral burr holes were drilled at coordinates based on the Paxinos and Watson brain atlas (relative to bregma: AP: 0.7, L: 3.4, V: 5.0) [16]. In the 6-OHDA lesion group, 3 μL of a solution (0.9% saline, 0.2% ascorbic acid, 5 μg 6-OHDA/μL) was injected bilaterally into the caudate-putamen at a rate of 0.5 μL/min. The injection cannula was held in place for 3 min before being carefully withdrawn to minimize reflux.

#### Parkinsonian Model Validation

Successful establishment of the parkinsonian phenotype was defined a priori using combined behavioral and histological validation criteria. Behavioral validation required the presence of reduced locomotor activity and impaired gait parameters in 6-OHDA-lesioned animals compared with healthy controls. Histological validation required a marked reduction in TH immunoreactivity in the SNc, indicating dopaminergic degeneration. Animals not demonstrating both predefined behavioral and histological lesion criteria were prospectively classified as exclusion candidates prior to analysis. However, all animals fulfilled these lesion success criteria and were therefore included in subsequent analyses.

### 2.2. Experimental Groups

**Figure 1:**
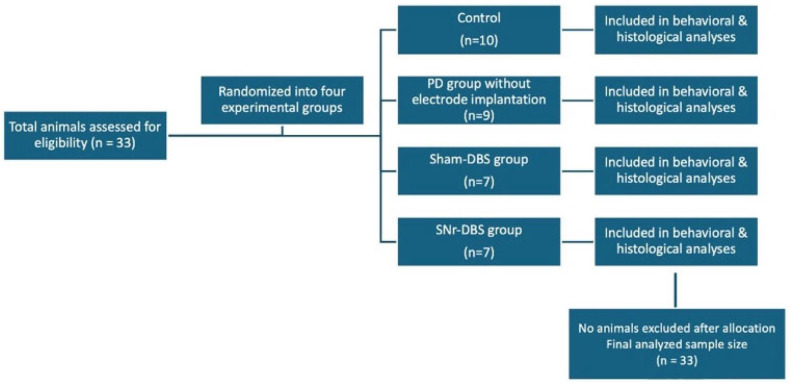
Animal flow diagram illustrating experimental allocation and analysis inclusion. A total of 33 rats were randomly assigned to four experimental groups prior to surgical procedures: Control (n = 10), PD (bilateral 6-OHDA lesion only; n = 9), PD + Sham-DBS (6-OHDA lesion with electrode implantation without stimulation; n = 7), and PD + SNr-DBS (6-OHDA lesion with active stimulation; n = 7). All animals completed the experimental protocol and were included in behavioral and histological analyses. No animals were excluded after group allocation. Final analyzed sample size (n = 33).

A total of 33 animals were included in the study. Rats were randomly assigned to four groups, including: (1) a healthy control group (n = 10); (2) a bilateral striatal 6-OHDA-lesioned PD group without electrode implantation (n = 9); (3) a sham-DBS group (n = 7) that underwent bilateral 6-OHDA lesioning and SNr electrode implantation without stimulation; and (4) an SNr-DBS group (n = 7) that received bilateral 6-OHDA lesions and active SNr stimulation applied for two weeks. All animals completed the experimental protocol and were included in the final behavioral and histological analyses. No animals were excluded following group allocation due to surgical complications, inadequate lesioning, or electrode misplacement confirmed by post mortem anatomical verification (Figure 1).

### 2.3. Electrode Placement in the SN and DBS Protocol

In the same surgical session as the 6-OHDA injection, bilateral concentric stimulating electrodes (Technomed, Beek, The Netherlands) were implanted into the SNr following burr hole creation. The electrodes, with a platinum–iridium inner cable, a 50 μm tip diameter, and a 250 μm shaft diameter, were positioned according to the Paxinos and Watson brain atlas (AP: −4.8, L: 2.5, V: −7 relative to bregma) [16]. After implantation, the electrodes were secured using dental cement, and the scalp was sutured. Animals were allowed to recover fully before the stimulation protocol began.

**Figure 2:**
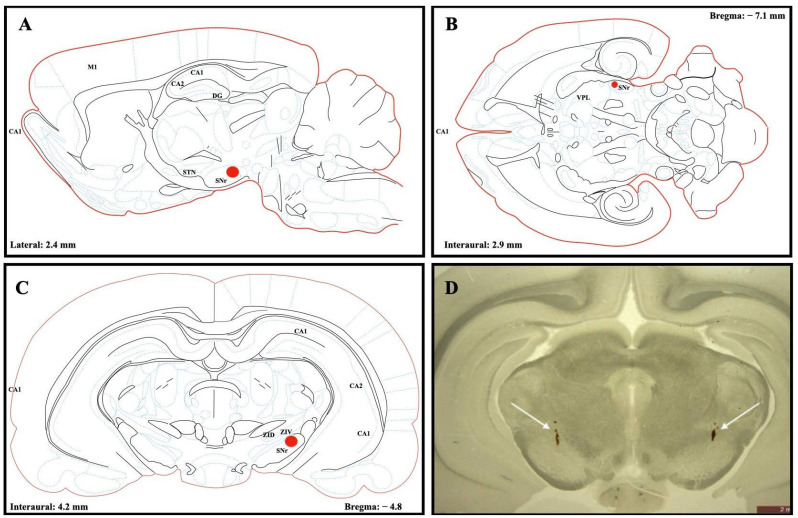
Histological section-based anatomical verification of electrode placement in the substantia nigra pars reticulata (SNr). Representative coronal brain section obtained after perfusion showing bilateral electrode tracks following stereotaxic implantation. Electrode tip localization was confirmed on histological sections relative to anatomical landmarks defined in the Paxinos and Watson rat brain atlas. All implanted animals demonstrated electrode trajectories consistent with accurate SNr targeting. (**A**) Sagittal, (**B**) horizontal, and (**C**) coronal illustrations of electrode tip locations based on stereotaxic coordinates and anatomical references. Red dots indicate targeted electrode tip positions based on post mortem anatomical identification of electrode tracks. Targeting coordinates for SNr implantation were AP: −4.8 mm, ML: ±2.5 mm, and DV: −7.0 mm, relative to bregma. (**D**) Representative coronal brain section obtained after perfusion demonstrating bilateral electrode tracks within the SNr (white arrows). Scale bar = 2 mm.

Electrode placement targeting the SNr was verified post mortem using coronal brain sections obtained after perfusion and fixation. Electrode trajectories and tip locations were anatomically identified on histological sections based on visible electrode tracks and confirmed relative to neuroanatomical landmarks defined in the Paxinos and Watson rat brain atlas. (Figure 2). Only animals demonstrating correct bilateral electrode placement within the SNr were included in the final analyses. Animals demonstrating off-target electrode placement outside the SNr were predefined as exclusion criteria prior to analysis; however, all implanted animals showed electrode trajectories consistent with accurate SNr targeting, and therefore no exclusions were required.

Deep brain stimulation was delivered using a WPI Acupulser A-310 stimulator coupled to an A-365 stimulus isolator (World Precision Instruments, Sarasota, FL, USA). Stimulation consisted of monophasic rectangular constant-current pulses delivered in cathodic polarity, with the implanted SNr electrode serving as the active (negative) electrode and the reference electrode functioning as the return pathway. High-frequency stimulation (130 Hz) with a pulse width of 60 µs was applied to the SNr, consistent with previous experimental studies [17]. Stimulation intensity was initiated at 20 µA to minimize stimulation-induced dyskinesia and increased in 20 µA increments at 1 min intervals to a final amplitude of 80 µA. Stimulation was administered for 3 h per day over 14 consecutive days.

Based on stimulation amplitude and pulse width, the maximum charge per phase was calculated as:

Q=I×PW=80 μA×60 μs=4.8 nC/phase

Assuming a circular electrode tip diameter of 50 μm (geometric surface area ≈ 1.96 × 10^−5^ cm^2^), the estimated maximum charge density was approximately 0.24 mC/cm^2^ per phase, remaining within established neural safety limits described by the Shannon safety model for electrical stimulation [18].

Electrode impedance was not directly measured during the experiment; however, stimulation was delivered in constant-current mode using an isolated stimulator, ensuring that variations in electrode–tissue impedance did not alter the delivered current amplitude. Concentric platinum–iridium microelectrodes of comparable geometry typically exhibit impedance values within the kilo-ohm range in brain tissue, which supports stable current-controlled stimulation in rodent DBS models. Together with charge density values remaining within established safety limits, these conditions indicate reliable and safe stimulation delivery throughout the experimental period [18].

The stimulation parameters were selected based on established experimental DBS paradigms in rodent models of PD. High-frequency stimulation at 130 Hz was chosen to reproduce clinically relevant DBS conditions known to suppress pathological basal ganglia activity and mimic therapeutic human DBS settings. Previous preclinical studies have employed a range of stimulation durations depending on experimental objectives, including short daily sessions and prolonged or continuous stimulation paradigms, all demonstrating behavioral and neurobiological effects of DBS in parkinsonian rodents [19,20,21]. The stimulation amplitude range (20–80 µA) was determined using stepwise escalation to identify effective current delivery while avoiding stimulation-induced adverse motor effects, consistent with prior rodent DBS methodologies using comparable electrode geometries [19]. Daily stimulation for 3 h over 14 consecutive days was therefore selected as an intermediate paradigm designed to model repeated therapeutic neuromodulation sufficient to induce neuroplastic and molecular adaptations while minimizing physiological stress and tissue burden associated with continuous stimulation protocols [21].

During the stimulation period, animals were monitored daily for procedure-related adverse effects including behavioral distress, abnormal motor activity, infection at the implantation site, or stimulation intolerance.

### 2.4. Behavioral Experiments

#### 2.4.1. Locomotor Activity

Locomotor activity was assessed using an open field system (MAY 9908 model, Commat Ltd., Ankara, Turkey) [22]. The system includes eight transparent cages with infrared photocells positioned at 4.5 cm and 11.5 cm above the floor, recording beam interruptions with 0.1 s sensitivity. Ambulatory activity is defined by complete positional changes, while horizontal activity refers to beam interruptions without location changes. Vertical movements, like rearing, are detected by upper photocells. The system calculates total locomotor activity and distance traveled. Each animal was placed in the center of the arena and recorded for five minutes. SNr-DBS was maintained throughout the test.

#### 2.4.2. Gait Analysis

Gait analysis was conducted 14 days post-electrode implantation using the footprint method. Rats walked on a 50 cm × 7 cm enclosed, paper-covered platform. Forelimbs were painted blue and hindlimbs red with non-toxic paint before testing. After 3 h of stimulation, three consecutive steps were analyzed. Key parameters included forelimb and hindlimb stride lengths (heel-to-heel distance) and stance width (distance between the first and fifth toes).

#### 2.4.3. Rotarod Test

Motor performance and coordination were evaluated using the rotarod test. Rats were acclimated to the device (Ugo Basile, Varese, Italy) for three days prior to testing. Thirty minutes before each test, they were placed in individual compartments facing the rotating rod (7.3 cm diameter). Time on the rod was recorded until the rat fell, with a maximum duration of 300 s. Performance was measured across speeds from 5 to 40 rpm, with a 5 min rest between speed changes to reduce stress and fatigue.

### 2.5. Histological Analyses

#### 2.5.1. Tissue Tracking for Paraffin Embedding

Rat brains were fixed in 10% formalin for 24 h, washed in water for 2 h, and dehydration was done with 70% and 80% alcohol for 48 h, 90% ethanol for 24 h, and 100% ethanol for 3 h. Tissues were cleared in xylene three times for 3 min each and embedded in paraffin at 56 °C.

#### 2.5.2. Immunohistochemical Labeling

Tyrosine hydroxylase expression in the SN, along with c-Fos and BDNF levels in the ventrolateral thalamus, cerebellar layers, and PFC, were assessed immunohistochemically. Due to the small size of the PPN, a suitable section for labeling could not be obtained.

For immunohistochemical analysis, 5 μm sections on superfrost slides were incubated at 56 °C overnight. Sections were deparaffinized in xylene for 10 min and passed through a descending alcohol series (100%, 90%, 80%, 70%) for 5 min each. After a 5 min wash in distilled water, antigen retrieval was performed by boiling in citric acid buffer (pH 6.0) at 750 W for 5 min, followed by cooling for 20 min. Sections were washed with PBS and blocked with UV blocking solution for 7 min. After blocking, sections were incubated overnight at 4 °C with anti-c-Fos (Abcam, Cambridge, UK #ab6167; 1/400), anti-BDNF (Abcam #ab46176), and anti-TH (Santa Cruz, Biotechnology, Dallas, TX, USA #sc-25269) antibodies. A negative control was included using an Ig-containing solution.

The following day, sections were washed with PBS and incubated with a biotin-labeled anti-rabbit secondary antibody (Vector, Laboratories, Burlingame, CA, USA #BA1000, 1/400) at room temperature for 1 h. After PBS washes, sections were incubated with streptavidin-peroxidase complex (Invitrogen, Carlsbad, CA, USA #85-9043) for 20 min, followed by Diaminobenzidine DAB (Sigma-Aldrich, St. Louis, MO, USA #D4168) substrate application for brown reaction evaluation. To enhance visibility, sections were counterstained with Mayer’s hematoxylin (Biooptica, Milan, Italy #06002L), dehydrated through an increasing alcohol series (70%, 80%, 90%, 100%), cleared in xylene, and sealed with Entellan (Merck, Darmstadt, Germany #1-07960-0500).

#### 2.5.3. ImageJ Analysis

At least six representative photomicrographs were obtained from each animal under identical acquisition settings. Quantitative assessment of immunoreactivity was performed using ImageJ software (version 1.50i, National Institutes of Health, Bethesda, MD, USA) by measuring optical density-based staining intensity within predefined regions of interest. Mean intensity values were calculated for each animal and used for group-wise statistical comparisons.

For quantitative validation of dopaminergic degeneration, TH immunoreactivity was analyzed using ImageJ software (National Institutes of Health, Bethesda, MD, USA)-based optical density measurements to compare dopaminergic marker expression between experimental groups. Because a bilateral 6-OHDA lesion model was employed, normalization to a contralateral hemisphere was not feasible; therefore, group-wise quantitative comparisons were used to evaluate lesion-associated dopaminergic depletion.

All image acquisition and quantification procedures were standardized to ensure reproducibility. Images were obtained using identical microscope settings, including illumination intensity, exposure time, and magnification, across all experimental groups. Regions of interest (ROIs) were anatomically predefined according to Paxinos and Watson atlas landmarks and they were applied consistently to all sections. Image analysis was performed using ImageJ software with identical threshold parameters applied across groups following background subtraction to minimize nonspecific staining variability. Quantification was conducted by an investigator blinded to experimental groups. For each animal, multiple anatomically matched sections were analyzed and averaged to obtain a single representative value per animal, and statistical analyses were performed using animal-level means.

### 2.6. Statistical Analysis

Statistical analyses were performed using the SPSS software (version 26.0,IBM Corp., Armonk, NY, USA). Data are expressed as mean ± standard deviation (SD). Normality of data distribution was assessed using the Shapiro–Wilk test. Comparisons between the experimental groups were conducted using one-way ANOVA followed by Tukey’s post hoc test for normally distributed data. For non-normally distributed data, Kruskal–Wallis test followed by Mann–Whitney U tests were applied. Pre-post analyses within groups were analyzed using paired *t*-tests or Wilcoxon signed-rank tests as appropriate.

Behavioral and histological outcomes were analyzed as predefined independent endpoints as they reflect distinct functional and biological domains, respectively. Accordingly, statistical analyses were performed separately for each outcome measure rather than using global models across endpoints. Post hoc corrections were applied within each analysis to control for multiple comparisons between experimental groups.

For rotarod testing, performance at different rotational speeds was analyzed separately given that increasing speeds represent progressively greater motor task difficulty rather than repeated longitudinal measurements of the same condition. Therefore, each speed was treated as an independent functional assessment consistent with common practice in preclinical motor behavior studies.

Given the predefined group-based experimental design and relatively small sample sizes typical of rodent DBS studies, one-way ANOVA-based comparisons were selected as a robust and appropriate analytical approach. More complex mixed-effects or factorial models were considered but were not implemented to avoid model overparameterization and instability in small experimental cohorts.

A *p*-value < 0.05 was considered statistically significant.

## 3. Results

Post mortem anatomical verification confirmed accurate bilateral SNr electrode localization in all implanted animals, with no off-target placements identified (Figure 2). Behavioral testing further confirmed successful induction of the parkinsonian phenotype in 6-OHDA-lesioned animals, demonstrated by significant reductions in locomotor activity and impaired gait parameters compared with healthy controls.

### 3.1. SNr-DBS Restored Activity

**Figure 3:**
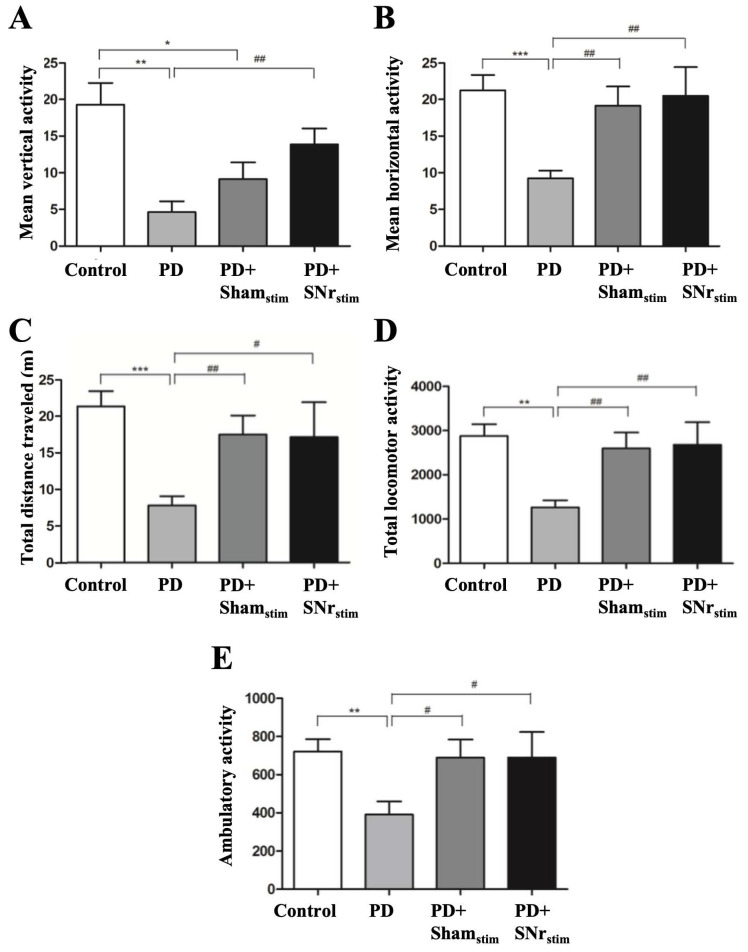
Locomotor activity test results: (**A**) mean vertical activity; (**B**) mean horizontal activity; (**C**) total distance traveled; (**D**) total locomotor activity; (**E**) mean ambulatory activity. Values are presented as mean ± standard deviation (SD). * *p* < 0.05, ** *p* < 0.01, *** *p* < 0.001: comparisons versus the control group; # *p* < 0.05, ## *p* < 0.01: comparisons versus the 6-OHDA group.

Locomotor activity was evaluated through vertical, horizontal, ambulatory activity, total locomotor activity, and total distance traveled. Parkinsonian rats showed significant reductions in all parameters compared to the healthy group (*p* < 0.01–0.001). In the sham-stimulation group, vertical activity increased, and significant improvements were observed in horizontal activity (*p* < 0.01), total distance (*p* < 0.01), total locomotor activity (*p* < 0.01), and ambulatory activity (*p* < 0.05) compared to the parkinsonian model. Notably, SNr-DBS further enhanced locomotor activity across all parameters, with significant increases in vertical (*p* < 0.01), horizontal (*p* < 0.01), total distance (*p* < 0.05), total locomotor activity (*p* < 0.01), and ambulatory activity (*p* < 0.05) compared to the parkinsonian model (Figure 3). Despite these improvements in the SNr-DBS group compared to the sham group, direct comparisons between the two groups did not reach statistical significance. This suggests that part of the locomotor recovery may reflect implantation-related effects, while SNr-DBS produced an additional, non-significant enhancement in the locomotor performance.

### 3.2. SNr-DBS Improved Gait Parameters

**Figure 4:**
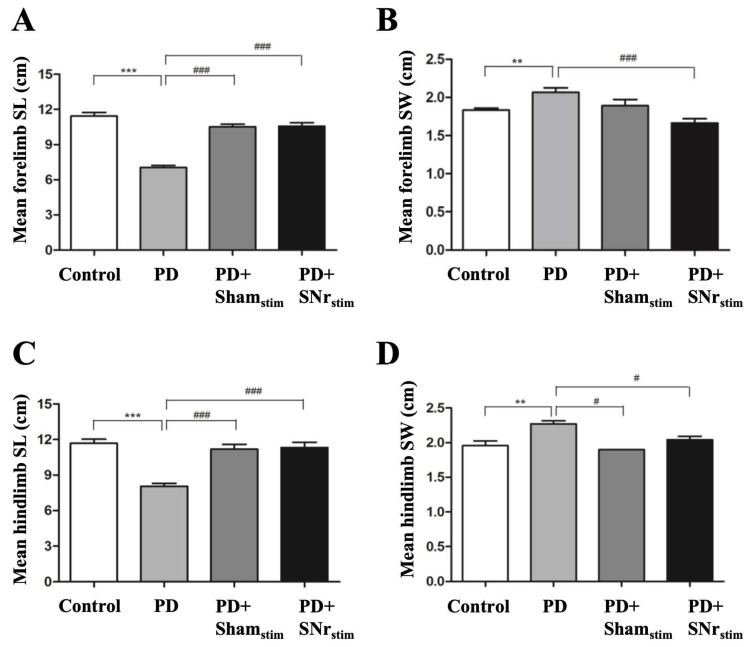
Footprint analysis results: (**A**) forelimb stride length; (**B**) forelimb stance width; (**C**) hindlimb stride length; (**D**) hindlimb stance width. ** *p* < 0.01, *** *p* < 0.001: comparisons versus the control group; # *p* < 0.05, ### *p* < 0.001: comparisons versus the 6-OHDA group.

Gait analysis using the footprint method assessed walking speed and stance width, focusing on forelimb and hindlimb stride length and stance width. In the parkinsonian group, both forelimb and hindlimb stride lengths were significantly reduced (*p* < 0.001), while stance widths were increased (*p* < 0.01). In the sham-stimulation group, stride lengths improved significantly (forelimb: *p* < 0.001, hindlimb: *p* < 0.001), and hindlimb stance width decreased (*p* < 0.05) compared to the 6-OHDA-induced parkinsonian group. SNr-DBS also enhanced gait parameters, significantly increasing forelimb (*p* < 0.001) and hindlimb (*p* < 0.001) stride lengths, and reducing stance widths (forelimb: *p* < 0.001, hindlimb: *p* < 0.05) compared to the parkinsonian group (Figure 4). Despite the further improvements in gait parameters in the SNr-DBS group compared to the sham-stimulation group, these differences did not reach statistical significance, suggesting that part of the gait recovery may be related to implantation-associated effects, while SNr-DBS provided an additional but non-significant improvement in gait performance.

### 3.3. SNr-DBS Tended to Enhance Balance and Coordination

**Figure 5:**
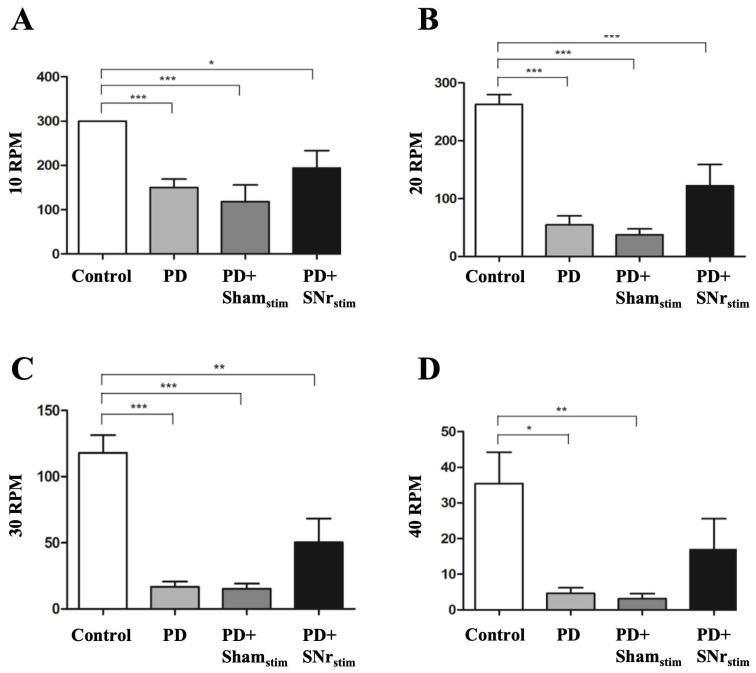
Rotarod performance results at different speeds: (**A**) 10 rpm; (**B**) 20 rpm; (**C**) 30 rpm; (**D**) 40 rpm. * *p* < 0.05, ** *p* < 0.01, *** *p* < 0.001: comparisons versus the control group.

The rotarod test, used to assess motor coordination and balance, measured the time spent on the device at speeds of 5, 10, 20, 30, and 40 rpm. Both parkinsonian and sham-stimulation groups showed significantly reduced latency to fall at 10, 20, 30, and 40 rpm compared to the healthy control group (*p* < 0.05–0.001), with no differences observed at 5 rpm, confirming motor impairment in the parkinsonian models. Although SNr-DBS demonstrated a trend toward improved motor coordination and balance compared to the sham-stimulation group, these improvements did not reach statistical significance at any speed (Figure 5).

### 3.4. SNr-DBS Restored Diminished TH Immunoreactivity in SNc

Histological and quantitative analyses confirmed successful induction of the parkinsonian phenotype. ImageJ-based optical density measurements demonstrated a marked reduction in TH immunoreactivity in the SNc of 6-OHDA-lesioned animals compared with healthy controls, consistent with dopaminergic degeneration.

**Figure 6:**
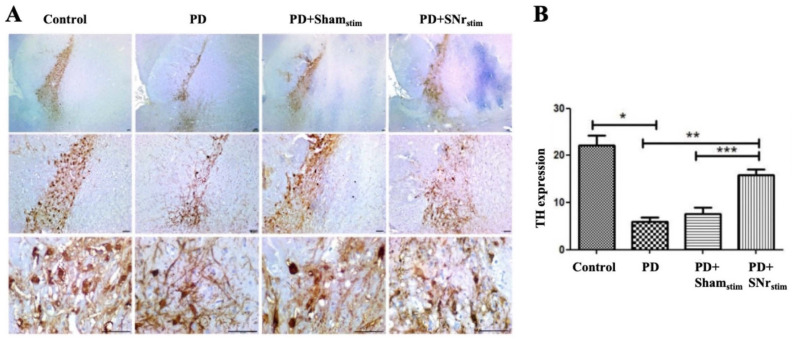
Tyrosine Hydroxylase (TH) immunoreactivity in the Substantia Nigra Pars Compacta (SNc): (**A**) representative images demonstrating differences in TH immunoreactivity in the SNc (4×, 10×, and 40× objective magnifications) across experimental groups. Strong TH immunoreactivity was observed in the control group, whereas reduced staining intensity was evident in the PD and PD+sham-stimulation groups. Importantly, TH immunoreactivity in the SNr-DBS group differed significantly from the sham-stimulation group, supporting a stimulation-specific biological effect beyond implantation-related changes. Scale bar = 50 µm. (**B**) quantitative analysis of TH immunoreactivity intensity measured using ImageJ software. Values are presented as mean ± SD. * *p* < 0.005 vs. control; ** *p* < 0.005 vs. 6-OHDA group; *** *p* < 0.005 vs. PD+sham-stimulation group.

Accordingly, TH expression was significantly reduced in the parkinsonian group compared to the healthy control group (*p* < 0.005). The sham-stimulation group showed similar TH expression levels, suggesting that electrode implantation alone did not confer a neuroprotective effect. In contrast, a significant increase in TH expression was observed in the SNr-DBS group compared to both the parkinsonian and sham-stimulation groups (*p* < 0.005 for both), indicating that SNr-DBS may partially restore dopaminergic marker expression and nigral dopaminergic integrity in the SNc (Figure 6).

### 3.5. SNr-DBS Reactivated c-Fos and BDNF Expression in Cerebellar Layers and Purkinje Cells in Parkinsonian Rats

**Figure 7:**
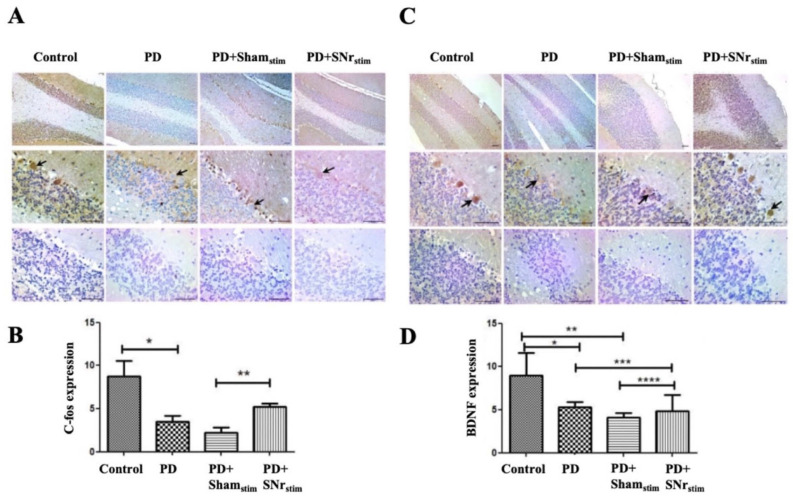
c-Fos and BDNF immunohistochemistry results in the cerebellum: (**A**) representative images show c-Fos expression intensity across groups (4×, 10×, and 40× objectives). Strong Purkinje cell expression is observed in controls, whereas expression is markedly reduced in the parkinsonian group (PD), with little to no reactivity in Purkinje cells (arrows) and preserved activity in adjacent basket cells. In the Parkinson + sham-stimulation (PD+Sham_stim_) group, Purkinje cell staining is decreased, with heightened basket cell reactivity. The PD + SNr stimulation (PD+SNr_stim_) group exhibits restored c-Fos expression in Purkinje cells and reduced basket cell activity, resembling the control profile. Bottom row: negative controls. Scale bar = 50 µm. (**B**) ImageJ-based quantification of c-Fos expression. * *p* < 0.005: comparisons versus the control group; ** *p* < 0.005: comparisons versus the PD+Sham_stim_ group. (**C**) representative images illustrate BDNF expression across groups (4×, 10×, and 40× objectives). Controls show robust expression, while parkinsonian rats exhibit reduced BDNF, limited to Purkinje cell membranes (arrows). PD+Sham_stim_ rats show similarly reduced staining, whereas PD+SNr_stim_ animals display increased BDNF in Purkinje cells. Bottom row: negative controls. Scale bar = 50 µm. (**D**) ImageJ analysis of BDNF expression. * *p* < 0.005: comparisons versus the control group; ** *p* < 0.001: comparisons versus the control group; *** *p* < 0.005: comparisons versus the PD group; **** *p* < 0.005: comparisons versus the PD+Sham_stim_ group.

c-Fos expression varied across cerebellar layers in the experimental groups. In control animals, c-Fos was observed in all layers, a pattern maintained in the DBS-treated groups. However, in the parkinsonian and sham-stimulated groups, c-Fos expression was significantly reduced, with Purkinje cells showing no expression, suggesting neuronal inactivity. SNr-DBS reactivated c-Fos expression, indicating a potential restoration of neuronal activity. ImageJ analysis revealed a significant reduction in c-Fos-positive cells in the parkinsonian and sham-stimulated groups compared to controls (*p* < 0.005). SNr-DBS markedly increased c-Fos expression in the cerebellar layers, reaching statistical significance only compared to the sham-stimulated group (*p* < 0.005) (Figure 7A,B).

Similarly, BDNF expression that was prominently observed across all cerebellar layers in the control group showed a significant reduction in both the parkinsonian and sham-stimulated groups (*p* < 0.005 for both). Importantly, SNr-DBS rescued BDNF expression in these groups (*p* < 0.005 for both), suggesting that DBS may offer protection against the loss of BDNF expression induced by 6-OHDA-related chemical damage (Figure 7C,D).

### 3.6. SNr-DBS Restored c-Fos and BDNF Expression in the Motor Cortex

**Figure 8:**
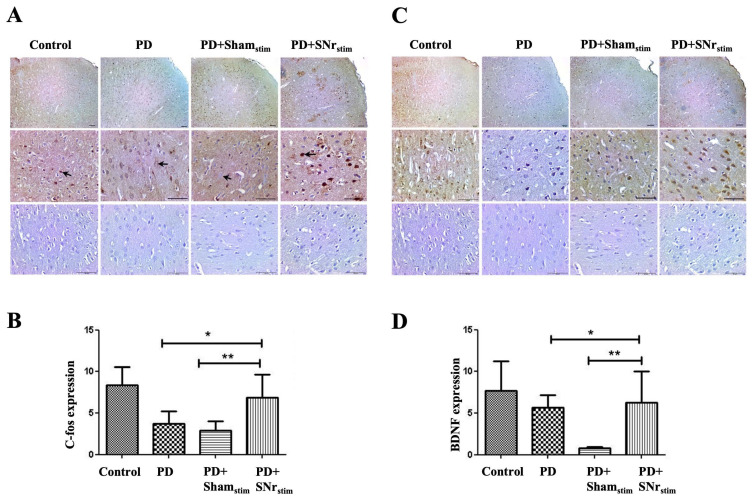
c-Fos and BDNF immunohistochemistry results in the frontal cortex: (**A**) representative images illustrate c-Fos expression intensity in the prefrontal cortex across groups (4×, 10×, and 40× objectives). In the control group, c-Fos expression is strong, while it is markedly reduced in the parkinsonian group (PD). In contrast, expression is restored in the PD + SNr stimulation (PD+SNr_stim_) group. The bottom row shows negative controls from the corresponding areas. Black arrows indicate c-Fos–positive nuclei. Scale bar = 50 µm. (**B**) ImageJ analysis showing group-wise differences in c-Fos expression. * *p* < 0.005: comparisons versus the PD group; ** *p* < 0.005: comparisons versus the PD+Sham_stim_ group. (**C**) representative images illustrate BDNF expression intensity in the prefrontal cortex across groups (4×, 10×, and 40× objectives). In the control group, BDNF expression is intense, whereas it is significantly markedly reduced in the PD group. In contrast, expression is restored in the PD+SNr_stim_ group. The bottom row shows negative controls from the corresponding areas. Scale bar = 50 µm. (**D**) ImageJ analysis of group-wise differences in BDNF expression * *p* < 0.05: comparisons versus the PD group; ** *p* < 0.005: comparisons versus the PD+Sham_stim_ group.

c-Fos immunoreactivity in the M1 and M2 regions of the frontal motor cortex was markedly reduced in both the Parkinson’s model and sham-stimulation group compared to healthy controls, though without statistical significance. Importantly, SNr-DBS resulted in a significant increase in c-Fos positivity compared to both the parkinsonian and sham-stimulated groups (*p* < 0.005 for each), suggesting that DBS normalizes c-Fos levels in these areas (Figure 8A,B).

BDNF expression mirrored the c-Fos findings, showing a marked reduction in both the Parkinson’s model and sham-stimulation group compared to controls. Notably, SNr-DBS led to significant restoration of BDNF expression in these groups (*p* < 0.005 for each), indicating a potential neuroprotective effect of SNr-DBS (Figure 8C,D).

### 3.7. DBS Recovered Diminished c-Fos and BDNF Expression in the Ventrolateral Thalamus

**Figure 9:**
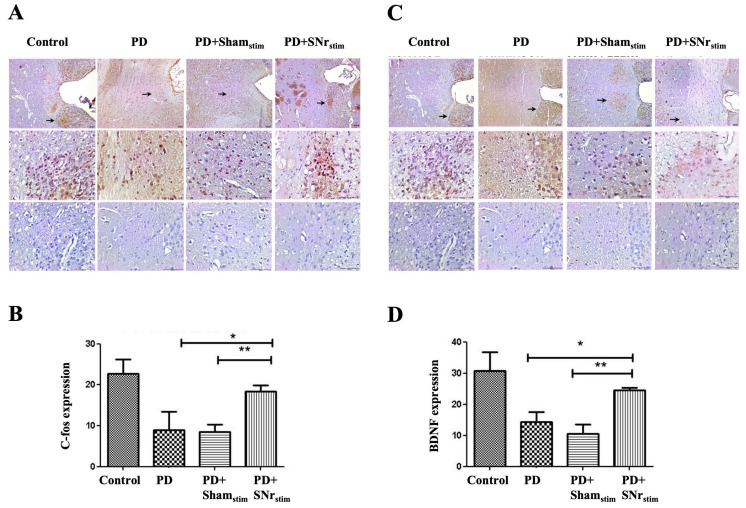
c-Fos and BDNF immunohistochemistry results in the ventrolateral thalamus: (**A**) representative micrographs depict c-Fos expression in the thalamic region across experimental groups (magnifications: 4×, 10×, 40×). In the control group, c-Fos expression is prominent, whereas in the parkinsonian (PD) group, it is significantly diminished. This reduction is reversed in the PD + SNr stimulation (PD+SNr_stim_) group, where expression levels appear elevated again. The bottom panel presents corresponding negative control images. Black arrows indicate c-Fos–positive nuclei. Scale bar = 50 µm. (**B**) quantitative analysis of c-Fos expression across groups based on ImageJ software. * *p* < 0.05: comparisons versus the PD group; ** *p* < 0.05: comparisons versus the PD+Sham_stim_ group. (**C**) representative images show BDNF immunoreactivity in the thalamus under 4×, 10×, and 40× objective lenses. While BDNF expression is robust in the control group, it is markedly reduced in the PD group. Notably, BDNF expression is re-elevated in the PD+SNr_stim_ group. Negative control sections for each region are displayed in the bottom row. Black arrows indicate BDNF-positive cells. Scale bar = 50 µm. (**D**) bar graphs illustrate group-wise BDNF expression intensity analyzed via ImageJ. (* *p* < 0.05: comparisons versus the PD group; ** *p* < 0.05: comparisons versus the PD+Sham_stim_ group.

c-Fos immunoreactivity in the ventrolateral thalamus was intense in the control group but reduced in the parkinsonian animals, including the sham-stimulation group, with preserved fiber staining to some degree, albeit with a marked decrease in nuclear staining. Notably, in the SNr-DBS group, the reaction closely resembled that of the control animals, suggesting a potential restorative effect of the stimulation. ImageJ analysis confirmed these findings, showing a significant recovery in c-Fos expression in the SNr-DBS group compared to both the parkinsonian and sham-stimulated rats (*p* < 0.05 for both) (Figure 9A,B).

Thalamic BDNF expression followed a similar pattern, with strong expression observed in the control group, a marked reduction in the parkinsonian group, and a re-emergence in the SNr-DBS group. ImageJ analysis further confirmed these findings, revealing a significant increase in BDNF expression in the SNr-DBS group compared to both the parkinsonian and sham-stimulated rats (*p* < 0.05 for both) (Figure 9C,D).

## 4. Discussion

The most important finding of this study is that 14-day SNr-DBS markedly enhanced locomotor activity in a 6-OHDA-induced PD model and showed a positive trend toward improving balance and motor coordination. Moreover, SNr-DBS partially restored TH-positive cells in the SNc and increased c-Fos and BDNF expression in the cerebellum, motor cortex, and thalamus, suggesting the first evidence of modulation of dopaminergic phenotype and functional integrity rather than direct evidence of neuronal survival. Based on the behavioral improvements observed in sham-stimulated animals, stimulation-specific effects should be interpreted cautiously, as implantation-related MLEs may partially contribute to functional recovery. Because electrode localization was verified post mortem on histological brain sections and all implanted animals demonstrated accurate SNr targeting, the observed behavioral and molecular effects can reasonably be attributed to stimulation of the SNr.

Surgical interventions have long been used in PD treatment, with lesioning causing tissue destruction and DBS modulating neural activity [23,24]; despite these differences, both approaches provide similar symptomatic relief. DBS inhibits neuronal activity by enhancing presynaptic GABA release, induces a depolarization block, and alters network dynamics by modulating firing rates and patterns [25]. It also influences neurotransmitter release, metabolism [26], neuroplasticity [27], the blood–brain barrier [28], and may have anti-neuroinflammatory effects [29].

The microlesion effect (MLE), or insertional effect, refers to the immediate motor symptom improvement following electrode insertion, occurring before active stimulation [30,31,32]. This transient phenomenon likely results from local micro-hemorrhages, edema, and neurotransmitter diffusion, temporarily altering neuronal activity and mimicking lesioning effects [33,34]. MLE has been proposed as an indicator of accurate electrode placement and a predictor of long-term DBS efficacy [35]. In this study, DBS-implanted groups showed improved locomotion and gait even without stimulation (sham-stimulation group), suggesting an early implantation-related MLE. Accordingly, behavioral improvements observed in sham animals are most consistent with an implantation-related MLE rather than neuromodulation induced by electrical stimulation, and therefore should be interpreted cautiously as reflecting surgical insertion effects rather than therapeutic DBS efficacy; consequently, implantation-related MLE may partially confound stimulation-specific outcomes. The pronounced MLE observed in the present study may also be related to the anatomo-functional characteristics of the SNr. Being the major inhibitory output nucleus of the basal ganglia, the SNr exerts tonic GABAergic control over downstream locomotor regions, particularly the mesencephalic locomotor region and pedunculopontine nucleus, that are known to be involved in locomotor functions such as gait and postural regulation. Even minor implantation-related mechanical disruption may hypothetically transiently reduce the pathological inhibitory output from the SNr, eventually resulting in partial disinhibition of locomotor circuits and behavioral improvement independent of the electrical stimulation itself. Such an insertion-related modulation may therefore be more pronounced when targeting basal ganglia output nuclei, providing a plausible explanation for the behavioral improvements observed in sham-stimulated animals in the present study. Additionally, immunohistochemical findings in the SNr-DBS group differed significantly from the sham group, supporting the hypothesis that DBS exerts its effects through ionic, protein, cellular, and network-level mechanisms [36].

The SNr, a major output nucleus of the basal ganglia, has been proposed as a DBS target [37,38,39] due to its role in motor control via connections with the mesencephalic locomotor region (MLR) [40]. Excessive GABAergic inhibition from the SNr is thought to impair MLR and pedunculopontine nucleus (PPN) functions, contributing to gait disturbances in advanced PD [41]. High-frequency SNr stimulation may counteract this by suppressing pathological PPN overactivity [38], potentially improving gait and posture. Clinical studies suggest that combined STN-SNr stimulation, targeting rostral and caudal contacts, could help treat freezing of gait [39,42]. However, findings on isolated SNr stimulation compared to STN remain inconsistent. One study reported that the former affected only temporal gait parameters, whereas the latter improved both spatial and temporal aspects [43]. Conversely, another study found SNr stimulation more effective in controlling anticipatory postural adjustments [44].

Building on these insights, research on SNr stimulation remains limited and conflicting between preclinical and clinical studies. In hemiparkinsonian rats, high-frequency SNr stimulation (150 Hz) reduced beta oscillations, decreased SNr neuronal spiking, and increased activity in the ventromedial thalamus, its primary efferent, while low-frequency stimulation (50 Hz) provided no benefit [45]. Clinically, however, SNr has been effectively stimulated at lower frequencies than STN [46], with combined low-frequency SNr and high-frequency STN stimulation improving gait in PD [47,48]. These findings suggest high-frequency SNr stimulation may inhibit GABAergic projections to the MLR, yet the optimal frequency and underlying cellular mechanisms remain unclear [46].

In this study, the parkinsonian group exhibited behavioral abnormalities, with changes in vertical and horizontal activity, ambulatory activity, total locomotor activity, and distance traveled during the locomotor activity test. Gait analysis showed reduced forelimb and hindlimb lengths, and increased stance width, consistent with previous rodent PD models [49]. Notably, SNr-DBS significantly improved locomotor activity and gait parameters, while sham stimulation also showed improvements, likely due to the MLE. Therefore, interpretation of stimulation-specific effects should primarily rely on comparisons between the SNr-DBS and sham-stimulation groups, which control for implantation-related MLE and isolate the contribution of active electrical stimulation. Although DBS had a greater impact than sham stimulation, statistical significance was not reached, possibly due to the study’s limited sample size. The rotarod test indicated impaired motor coordination and balance in the parkinsonian group, with SNr-DBS showing a tendency to improve performance across all speeds, but without statistical significance. Based on these findings, our results suggest that SNr-DBS has a positive effect on locomotor activity and gait parameters, supporting its potential as an effective therapeutic approach for improving motor function in parkinsonian models. Importantly, these interpretations should be considered associative rather than strictly causal, given the contribution of insertion-related effects observed in sham-stimulated animals.

Tyrosine hydroxylase, the rate-limiting enzyme in dopamine synthesis, is a key marker of dopaminergic integrity, often used to assess neuron loss in PD models [50,51]. While STN-DBS has been shown to protect TH-positive nigrostriatal neurons in PD [20,52], the impact of SNr-DBS on dopaminergic neuron survival remains unclear. In this study, we observed a significant reduction in TH-positive cells in the SNc following 6-OHDA treatment. Notably, after 14 days of SNr-DBS, TH immunoreactivity significantly increased. While TH expression alone cannot directly demonstrate dopaminergic neuron survival, these findings suggest modulation of dopaminergic phenotype and functional integrity following stimulation.

We also assessed c-Fos and BDNF levels as markers of neuronal activation and neuroprotection in the cerebellum, PFC, and thalamus for several reasons. c-Fos is an immediate-early gene reflecting transient neuronal activation rather than a permanent structural or neuroprotective cellular change; c-Fos findings of the present study should therefore be interpreted as indicators of stimulation-associated network engagement. First, the cerebellum plays a role in both PD pathophysiology and compensation [53,54,55], with reduced volume observed in imaging studies [56], though no significant neuronal loss or α-synuclein accumulation is detected [57]. Though previously considered separate, recent evidence highlights anatomical and physiological connections between the cerebellum and basal ganglia [54], suggesting their joint influence on posture and gait through brainstem and cortical circuits [58]. Second, the PFC is involved in two cholinergic pathways affecting mobility: one from the nucleus basalis of Meynert projecting to the cortex, including the PFC, and another from the PPN, which projects to multiple regions, including the thalamus and cerebellum [59]. Lastly, dopamine depletion in PD disrupts thalamic regulation by enhancing striatal inhibition of the globus pallidus externus, leading to STN-driven hyperactivity and motor deficits [60,61]. Our findings showing increased BDNF and c-Fos expression after SNr-DBS align with evidence that DBS induces widespread biological effects beyond local circuit modulation. Supporting this, Kocabıcak et al. (2025) [62] demonstrated that STN-DBS alters peripheral cytokine levels in PD patients, indicating systemic immunomodulatory actions of DBS. Together, their clinical results and our experimental data suggest that DBS regardless of the target structure may exert therapeutic benefits through broader neuroplastic and neuroimmune pathways, underscoring the relevance of SNr as a potential DBS target [62]. Consistent with these findings, our study demonstrated decreased neuronal activity in the cerebellum, PFC, and thalamus of parkinsonian rats, as indicated by reduced c-Fos immunoreactivity. Notably, SNr-DBS significantly restored neuronal activity in these regions, offering new evidence that SNr-DBS may counteract PD-related disruptions in these interconnected structures.

Brain-derived neurotrophic factor, a neurotrophin crucial for neuronal differentiation, survival, and neurogenesis, exhibits neuroprotective effects under stress [63]. BDNF promotes dopaminergic neuron survival, dopamine release, and turnover in the striatum, and reduced levels are implicated in neurodegenerative diseases like PD [64]. After STN-DBS, increased BDNF levels were observed in the nigrostriatal system, M1 cortex, and GPi, suggesting a potential neuroprotective role of STN-DBS, partly mediated by BDNF [21]. Preclinical studies also indicate that DBS induces neuroprotection in 6-OHDA-induced parkinsonism through activation of tropomyosin receptor kinase B (TrkB), a key downstream effector of BDNF [65]. Furthermore, STN-DBS has shown potential disease-modifying effects by restoring disrupted BDNF transport in nigrostriatal and corticostriatal circuits affected by pathological α-synuclein inclusions [66]. In our study, BDNF expression was significantly reduced in the cerebellum and showed a non-significant decrease in the PFC and thalamus. However, SNr-DBS significantly increased BDNF levels across all three regions, providing evidence suggestive of neuroprotective-associated molecular changes in a neuroprotective effect of SNr-DBS and reinforcing its potential as a therapeutic target in PD.

Despite the promising findings, several limitations should be considered when interpreting the results of this study. First, the relatively small sample size may have limited the statistical power, particularly in detecting significant differences between the DBS and sham groups in certain behavioral and histological outcomes. Additionally, the short-term 14-day stimulation period does not allow for an assessment of the long-term effects of SNr-DBS, and further studies are needed to evaluate its chronic impact. While SNr-DBS demonstrated improvements in locomotor activity, gait, and motor coordination, the exact cellular mechanisms underlying these changes warrant further investigation.

Although quantitative assessment of TH immunoreactivity was performed using optical density-based ImageJ analysis, stereological TH-positive neuron counting or striatal fiber density measurements could provide additional structural quantification. However, the present study employed a bilateral 6-OHDA lesion model, in which behavioral impairment together with consistent reductions in TH immunoreactivity jointly confirmed successful induction of the parkinsonian phenotype. Therefore, TH findings should be interpreted as reflecting alterations in dopaminergic marker expression and functional integrity rather than direct measurements of neuronal survival.

Furthermore, although the high-frequency stimulation applied in this study (130 Hz) is consistent with previous literature, clinical studies suggest that lower-frequency SNr-DBS may have different therapeutic effects. Our study did not investigate the impact of lower-frequency stimulation on locomotor activity and gait, leaving this aspect unexplored. Another limitation is that our analysis primarily focused on locomotion and gait parameters, while PD encompasses a broader spectrum of symptoms, including cognitive, affective, and non-motor disturbances, which were not assessed in this study. Lastly, the observed microlesion effect in the sham-stimulation group may confound the interpretation of DBS-specific effects, as it highlights the immediate impact of electrode implantation. Stimulation-related effects should therefore be concluded with caution and viewed as reflecting stimulation-associated changes within the constraints of the experimental design of the present study. Future studies with larger sample sizes, longer stimulation durations, and broader symptom assessments are needed to further clarify the potential of SNr-DBS as a comprehensive therapeutic strategy for PD.

## 5. Conclusions

In this study, we provide evidence supporting the potential neuroplastic and dopaminergic modulatory effects in PD. Our findings demonstrate that 14-day SNr stimulation improved locomotor activity, gait parameters, and motor coordination in a 6-OHDA-induced rat model of PD, with notable improvement of dopaminergic marker expression and neuronal activity in key brain regions. The observed increase in BDNF expression further suggests a potential role of SNr-DBS in modulating processes associated with neurodegeneration, offering promising insights into its therapeutic potential. While further research is necessary to refine stimulation protocols and elucidate the underlying cellular mechanisms, SNr-DBS emerges as a viable target for future PD treatments, potentially complementing existing therapies to enhance motor function and slow disease progression.

## Data Availability

The data underlying this article are available in this article. If needed, please contact the corresponding author. The email address is pinarocak@uludag.edu.tr.

## Abbreviations

The following abbreviations are used in this manuscript:

6-OHDA	6-Hydroxydopamine
BDNF	Brain-derived Neurotrophic Factor
DBS	Deep Brain Stimulation
GPi	Internal Globus Pallidus
PFC	Prefrontal Cortex
PD	Parkinson’s Disease
SNc	Substantia Nigra Pars Compacta
SNr	Substantia Nigra Pars Reticulata
STN	Subthalamic Nucleus
TH	Tyrosine Hydroxylase
